# Eribulin Efficacy on Brain Metastases in Heavily Pretreated Patients with Metastatic Breast Cancer

**DOI:** 10.3390/jcm10061272

**Published:** 2021-03-18

**Authors:** Renaud Sabatier, Johan Martin, Cécile Vicier, Mathilde Guérin, Audrey Monneur, Magali Provansal, Louis Tassy, Carole Tarpin, Jean-Marc Extra, Frédéric Viret, Anthony Goncalves

**Affiliations:** 1 Department of Medical Oncology, Institut Paoli-Calmettes, 13009 Marseille, France; MARTINJ@ipc.unicancer.fr (J.M.); VICIERC@ipc.unicancer.fr (C.V.); GUERINM2@ipc.unicancer.fr (M.G.); MONNEURA@ipc.unicancer.fr (A.M.); PROVANSALM@ipc.unicancer.fr (M.P.); TASSYL@ipc.unicancer.fr (L.T.); TARPINC@ipc.unicancer.fr (C.T.); EXTRAJM@ipc.unicancer.fr (J.-M.E.); VIRETF@ipc.unicancer.fr (F.V.); GONCALVESA@ipc.unicancer.fr (A.G.); 2 Aix-Marseille Univ, CNRS U7258, INSERM U1068, Institut Paoli-Calmettes, CRCM, 13009 Marseille, France

**Keywords:** metastatic breast cancer, brain metastasis, eribulin

## Abstract

The onset of brain metastases (BM) is a major turning point during advanced breast cancer (ABC) evolution, with only few treatment options when local therapies have failed. The therapeutic effect of eribulin, a wildly used drug in the treatment of ABC, remains unclear in this setting. Patients and Methods: We performed a retrospective observational study to assess eribulin efficacy in patients with ABC who displayed BM at time of eribulin initiation. We collected data from the medical files of all ABC patients who received eribulin at our institution from 2012 until 2020. Our main endpoint was the central nervous system (CNS) progression-free survival. (CNS-PFS). Other evaluation criteria were extra-cranial progression free survival (PFS) and overall survival (OS). Results: Twenty patients with BM monitoring data available were selected out of the 549 who received eribulin during the inclusion period. Fifteen patients (75%) had BM progressive as the best response, three patients (15%) had disease stabilization for more than 6 months and only one patient had a partial response according to RECIST 1.1 criteria. Median CNS-PFS was 3.39 months (95CI (3.02–3.76)). Cox univariate analysis identified molecular subtype as the only prognostic parameter in our cohort, with patients with hormone-receptor positive tumors less likely to experience CNS progression than those with triple-negative MBC (HR = 0.23 (95CI = 0.07–0.80), *p* = 0.021). Median extra-cranial PFS was 2.67 months (95CI (2.33–3.01)). Median OS was 7.68 months (95CI (0–17.41)). Conclusion: Eribulin seems to have only a limited impact on BM evolution. Hormone receptors expression may identify a subset of patients with better BM control.

## 1. Introduction

The rising incidence of breast cancer brain metastases (BCBM) is a major clinical problem with a prevalence of up to 30%, 80% of which have simultaneous extra-cranial disease. These patients have a poor prognosis with a median survival time ranging from 5 to 13 months [1]. Moreover, recent gains related to new therapies developed for extra-cranial disease have dramatically improved the overall survival of patients with metastatic breast cancer (MBC). As BCBM are often diagnosed late during MBC natural history, BCBM incidence is growing. As the prognosis remains very poor and efficient systemic therapies are scarce, BCBM are a clear unmet medical need.

Standard treatment for BCBM is mainly based on local therapies, including whole brain radiotherapy (WBRT) or stereotactic radiation surgery (SRS) and, in selected cases, neurosurgery. While these local treatments may significantly reduce brain metastases progression rates, there is no clear evidence of an effect on survival, and patients may be at risk of serious quality-of-life altering adverse effects, including cognitive deficiencies [2]. WBRT+SRS, for a limited number of brain metastases, is associated with significantly worse cognitive function than SRS alone and no overall survival benefit despite better tumor disease control [3]. SRS has thus become the standard of care for patients with limited brain disease. In case of disease progression after SRS and/or WBRT, the therapeutic options available are limited, and a systemic treatment may be administered with limited efficacy [4].

Eribulin mesylate is a synthetic analogue of the marine natural product halichondrin B that inhibits microtubule dynamics by binding to the vinca domain on tubulin [5,6]. In randomized controlled studies [7,8,9], eribulin improved survival in HER2-negative, taxanes- and anthracyclines-pretreated metastatic breast cancer. However, most of the patients with brain metastases were excluded from these trials, and eribulin efficacy for BCBM remains unclear.

Even though eribulin does not cross the healthy blood brain barrier [10], its efficacy on CNS disease can be enhanced if the blood brain barrier is disrupted by metastases outgrowth and/or after cranial radiation therapy. Exploratory analysis of the Study 301 showed that BCBM were observed for only 2.4% of patients in the eribulin arm versus 4.6% in the capecitabine arm [8]. Some case reports were published suggesting that eribulin may have a higher protective effect on BCBM than other standard chemotherapies [11,12,13]. 

We developed this retrospective to add new data related to eribulin efficacy on BCBM. Our main objective was to assess the central nervous system progression-free survival (CNS-PFS) of patients treated for BCBM, and to identify clinicopathological features associated with eribulin efficacy in this setting. 

## 2. Patients and Methods

### 2.1. Patient Selection and Data Collection

We conducted this monocentric retrospective study among the population of the Institut Paoli Calmettes (IPC, Marseille, France), analyzing the electronic medical files of all the patients diagnosed with MBC who received eribulin from April 2012 (date of eribulin approval in Europe) until August 2020, and for whom efficacy data could be obtained. Follow-up data collection was stopped on August 31, 2020. The patients received eribulin at the starting dose of 1.23 mg/m^2^ at day 1 and day 8 of 21-day cycles until disease progression or unacceptable toxicity. Performance status was defined by the Eastern Cooperative Oncology Group (ECOG) score. 

Because this was a retrospective non-interventional study, formal informed consent was not required. This work was performed after approval from our institutional review board (IPC Groupe de Selection des Essais Cliniques). All procedures were done in accordance with French ethical standards.

### 2.2. Endpoints and Assessments

The primary aim was to evaluate the efficacy (PFS and OS) of eribulin in this setting. Our main endpoint was CNS-PFS. We also explored extra-cranial PFS, whole PFS and overall survival (OS). 

Treatment efficacy was evaluated by conventional Response Evaluation Criteria in Solid Tumors (RECIST 1.1) criteria [14] every four cycles (usually 12 weeks), or whenever clinically indicated. Stable disease or response had to be confirmed in the subsequent tumor evaluation. PFS was defined as the time from the first eribulin cycle until objective tumor progression or death from any cause. CNS-PFS was defined as the time from the first eribulin cycle until objective BCBM progression or death from any cause. Extra-cranial PFS was defined as the time from the first eribulin administration until objective extra-cranial tumor progression or death from any cause.

OS was defined as the time from the first eribulin cycle to death from any cause. We also described the rates of complete and partial responses, as well as stable disease and progressive disease according to RECIST 1.1 criteria. Clinical benefit rate was defined as the rate of patients with an objective response or with disease stabilization for more than 6 months.

CNS evaluation was based on brain MRI performed every three months or more often, if clinically indicated, for patients known to have BCBM at eribulin initiation. Brain imaging was not performed systematically for patients with ABC treated by eribulin without prior history of BCBM, as it is recommended by international guidelines.

### 2.3. Statistical Analyses

Categorical variables were described using counts and frequencies, and quantitative variables were described using medians and ranges. PFS and OS rates were estimated using the Kaplan–Meier method and two-sided 95% confidence intervals (95CI) were presented. Patients without progression or death were censored at the date of last news. Correlations between clinicopathological features and survival were performed using a univariate Cox regression model, and the significance of the coefficients correlated to each parameter was assessed using the Wald’s test. Statistical analyses were carried out with the SPSS^®^ software version 17 (IBM™, Armonk, NY, USA). We followed the reporting recommendations specified in the STROBE (Strengthening the Reporting of Observational Studies in Epidemiology) Statement [15] (Table S1). 

## 3. Results

### 3.1. Patients

Twenty patients receiving eribulin for MBC with previously diagnosed BCBM were identified among the 549 patients who received eribulin for MBC in our comprehensive cancer center during the inclusion period (Figure S1—Flowchart). Clinical and pathological features are described in Table 1. The median age was 56.4 years (range, 29.7–74.3). ECOG performance status was 1 for 75% of patients and two or more for 25%. Two patients had a history of confirmed carcinomatous meningitis. Half of our patients had HR+ disease (including two HR+/HER2+), 25% were HER2-positive, and 35% displayed a triple-negative cancer.

Most patients (75%) received previous BCBM therapies before inclusion, including SRS (*n* = 5), neurosurgery (*n* = 4), and WBRT (*n* = 8). Two of them received two of these treatments. Patients received a median of four (range, 1–8) previous systemic therapies for MBC. Patients included in this cohort received a median of one line of chemotherapy (range, 0–5) after eribulin failure. None of the patients was still on treatment on the date of the follow-up data cut-off.

### 3.2. Eribulin Efficacy on BCBM

**Figure 1:**
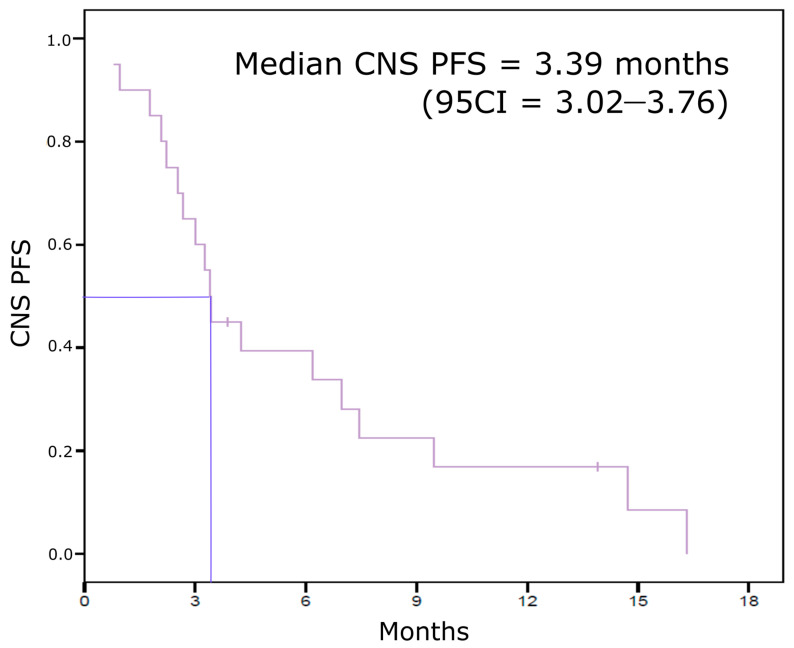
Central nervous system progression-free survival (CNS-PFS) Kaplan–Meier curve.

Eribulin was not effective on BCBM for most of the patients included in our cohort, with 75% progressive CNS disease as the best response. Three patients (15%) had a disease stabilization for more than 6 months and we observed BCBM response for only one patient according to RECIST 1.1. The four patients who displayed a CNS benefit from eribulin had HR+/HER2- tumors. Median follow-up was 7.68 months. Median CNS-PFS was 3.39 months (95CI (3.02–3.76)) (Figure 1). BCBM were still controlled more than 1 year after eribulin initiation for three patients. 

Cox univariate analysis identified molecular subtype as the only prognostic parameter in our cohort (Table 2), with patients with HR+ tumors less likely to experiment CNS progression than those with triple-negative MBC (*p* = 0.021, HR = 0.23 (95CI = 0.07–0.80)). Age and PS at eribulin initiation, the number of previous systemic lines for MBC, and previous CNS therapies were not associated with CNS disease evolution (Table 2). As brain radiation and neurosurgery have been shown to modify cytotoxic drugs capacity to penetrate through the blood–brain barrier [16], we explored if previous CNS treatment could modify eribulin efficacy on BCBM. Previous CNS therapies did not improve CNS PFS (*p* = 0.38, HR = 0.62 (95CI = 0.21–1.80)). This result was similar when comparing WBRT to SRS or neurosurgery.

### 3.3. Other Survival Endpoints (Extra-Cranial Response, Extra-Cranial PFS, Whole PFS, and OS)

**Figure 2:**
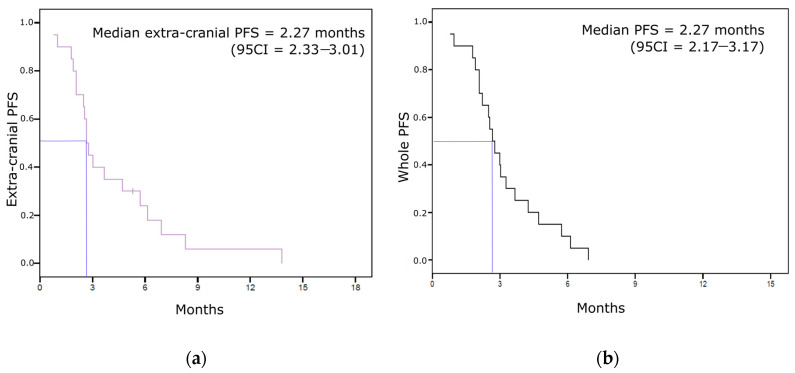
Non-CNS PFS Kaplan–Meier curves. (**a**) Extra-cranial progression-free survival. (**b**) Whole progression-free survival.

We had body CT-scan available for 19 of the 20 patients included in the BCBM analysis. Most of them (16/19, 84%) experienced extra-cranial disease progression as best response, two (11%) had stable disease and only one (5%) displayed a partial response of her extra-cranial metastases. Concerning the four patients with BCBM control with eribulin, three of them had early extra-cranial disease progression (one breast tumor progression and two liver metastases progression). Only one patient had both CNS and extra-cranial response under treatment, but with a 6.9-month time from treatment initiation to bone progression versus 14.7 from baseline to CNS progression. Median extra-cranial PFS was 2.67 months (95CI = 2.33–3.01). All but four patients had extra-cranial progression within 6 months after the first eribulin administration (Figure 2a). Whole PFS was 2.67 months (95CI = 2.17–3.17) (Figure 2b). All patients experimented disease progression (whatever the site of progression) within seven months after eribulin initiation (longest PFS was 6.9 months).

**Figure 3:**
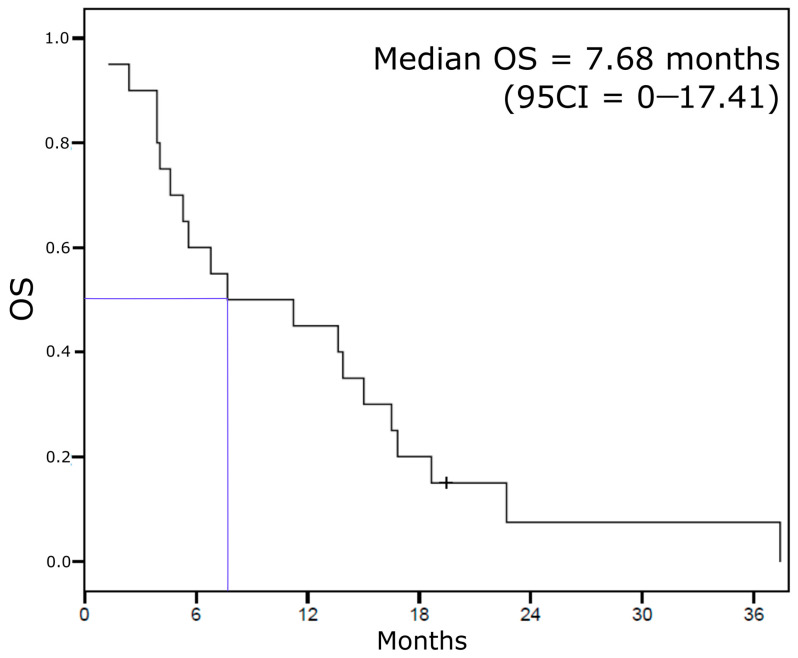
Overall survival Kaplan–Meier curve.

Median overall survival was 7.68 months (Figure 3). Nine patients lived longer than 1 year, including one outlier who died 37 months after eribulin initiation. It is of note that this patient had the longest CNS-PFS (16.3 months). ECOG PS was the only clinicopathological feature at inclusion associated with OS (Table 3). HR expression and a triple-negative phenotype were not associated with OS.

## 4. Discussion

We show in this retrospective study that only a small subset of patients treated with eribulin was previously diagnosed with BCBM. We also observed that eribulin efficacy on BCBM was limited to HR+/HER2- tumors, and that the outcomes in this setting were close to that described in randomized trials exploring eribulin efficacy for patients without CNS disease.

Whole PFS and OS were shorter than what was described in randomized phase III trials, but also than what was shown in large real-world cohorts [17,18]. In a large meta-analysis published in 2016, median PFS was 3.9 months for the eribulin arm vs. 3.2 months for the control group, and OS was 15.0 vs. 12.6 months [9]. Median whole PFS and OS were 2.7 months and 7.7 months in our cohort, respectively. This can be explained by the higher number of previous treatment lines in our set with a median of four lines, whereas patients included in the Study 301 should have received no more than three treatment regimens for MBC [8]. In the same way, thirty percent of the patients included in the meta-analysis received only one line of chemotherapy before eribulin initiation versus only one of 20 in our cohort. Our results are also consistent with the well-described poorer outcomes of BCBM [19,20]. 

CNS PFS and extra-cranial PFS were close in the current study (3.4 vs. 2.7 months). This is consistent with the results of a small retrospective cohort of 66 patients including 19 with BCBM [21]. Efficacy analysis of eribulin in various subgroups showed that patients with BCBM had similar outcomes when compared to other visceral metastatic sites.

The efficacy of systemic therapies on brain tumors is correlated to their abilities to cross the blood brain barrier (BBB) and to avoid efflux mechanisms [22]. The BBB of brain metastases was described to be more permeable than that of primary brain tumors [23]. Moreover, this permeability may also be increased because of low P-glycoprotein expression in brain metastases [24]. BBB permeability can be also increased by radiation therapy, and radiotherapy-chemotherapy combinations may be of interest for these patients [25,26]. Efficacy of various radiation methods (WBRT versus SRS) was not described previously in this setting, even if some scarce data from case reports were published concerning combinations of eribulin and WBRT [13,27,28]. We observed in this cohort that only a small subset of our patients (20%) presented a CNS clinical benefit with eribulin in this setting, all had HR+/HER2- tumors. As it was already published, CNS-PFS was decreased for HR- cases [29,30]. This suggested that eribulin may not be a priority option for BCBM of other phenotypes, and that BCBM should be screened for triple-negative cases to enable focal treatments such as SRS and surgery instead of systemic therapies [31]. 

However, we also observed no OS benefits through molecular subtypes. Treatments based on HER2-inhibitors showed a dramatic efficiency on BCBM of HER2+ diseases, with the greatest improvement obtained using tyrosine kinase inhibitors lapatinib, neratinib pyrotynib, and tucatinib [32,33,34,35]. In the HER2CLIMB randomized phase III trial, the combination of tucatinib with trastuzumab and capecitabine for patients with trastuzumab-resistant MBC showed a 68% reduction of the risk of brain metastases progression or death (hazard ratio 0.32; 95% CI (0.22–0.48); *p* < 0.0001) [34]. For HER2- tumors, because clinical trials have often excluded patients with BCBM, we only have scarce retrospective data. Some responses were observed with traditional cytotoxic drugs such as platinum [36]. Other chemotherapies, including taxanes, are not effective in this setting because they cannot cross the BBB and have low brain and CSF concentrations. For these tumors, no data exist concerning endocrine therapies clinical activity. However, new targeted therapies such as PARP-inhibitors, PI3K inhibitors, and CDK4/6 inhibitors are under investigation in this setting [36]. The effect of eribulin for HR+/HER2- tumors (four with clinical benefit out of 8 cases) in our cohort suggested that there may be a place for this drug in this context. Compared to other chemotherapies used for endocrine-resistant tumors, eribulin administration should be discussed after anthracyclines and taxanes failure.

Our work has limitations. First, its small sample size and its retrospective design prevented us to affirm a high-level of evidence-based conclusions. However, this can be explained by the low incidence of diagnosed BCBM in the population of patients with MBC candidate to eribulin therapy. We observed only 10.9% (*n* = 60) patients with BCBM among the 549 patients who received eribulin in this setting in our institution. No prospective trial seems to be able to be developed in such a rare clinical situation. Then, our population was heterogeneous with triple-negative, HR+, and HER2+ MBC. This may have decreased our capacity to identify the impact of eribulin in each of these subgroups. This could have been of interest as breast cancer molecular subtypes can display very different profiles of response to systemic therapies. However, the small sample size limits vastly the relevance of this exploratory analysis. As not all potential confounders can be ruled out, eribulin value in each subtype remains preliminary. Nevertheless, CNS-PFS was longer for pretreated HR+ cases (HR = 0.23 vs. triple negative tumors), suggesting that there may be a signal for eribulin impact on BCBM in this subtype. The heterogeneity of our dataset also limits data interpretation according to the number of prior lines of therapies. However, Cox regression analyses did not find any PFS or OS discrepancy depending on this parameter. Finally, the retrospective design did not allow us to analyze clinical features such as clinical status variations under treatment and steroid use before and under treatment. These features are included in the Response Assessment in Neuro-Oncology Brain Metastases (RANO-BM) criteria that are now a standard for systemic therapies assessment for the treatment of brain metastases in clinical trials [37]. Combining radiological and clinical features for treatment efficacy assessments could indeed be of high clinical value as brain metastases can impair the functional and cognitive functions of our patients.

## 5. Conclusions

In conclusion, we described here the largest cohort of patients receiving eribulin for MBC and who displayed BCBM before treatment initiation. We showed that survival was poorer in this dataset comparing to randomized trials evaluating eribulin that excluded patients with BCBM, and that a poor performance status and a triple-negative phenotype were correlated to even worse CNS outcome with eribulin. 

## Figures and Tables

**Table 1:** Clinical and pathological features at inclusion. When required, results are notified as *n* (% of cases with data available).

		*n* = 20
Age at inclusion, years (median, min-max)		56.4 (29.7–74.3)
Performance status at eribulin initiation	1	15 (75)
	≥2	5 (25)
Previous brain metastases treatments	None	5 (25)
	Whole brain radiotherapy	8 (40)
	Stereotactic surgery	5 (25)
	Brain neurosurgery	4 (20)
Carcinomatous meningitis		2 (10)
Priori systemic lines of treatment for MBC (median, min-max)		4 (1–8)
HR status	Positive	10 (50)
	Negative	10 (50)
HER2 status	Positive	5 (25)
	Negative	15 (75)
Triple negative phenotype		7 (35)
Number of lines post-eribulin (median, min-max)		1 (0–5)

HR: hormone receptors; MBC: metastatic breast cancer.

**Table 2:** Central nervous system (CNS) progression-free survival (PFS) cox regression univariate analysis.

CNS PFS Cox Univariate Analysis (*n* = 20)		Hazard Ratio (95CI)	*p*-Value
Age at inclusion, years		0.98 (0.95–1.02)	0.30
ECOG PS	1 vs. ≥2	0.47 (0.14–1.60)	0.23
Pathological subtype			0.03
	HR+ vs. TN	0.23 (0.07–0.80)	0.02
	HER2+ vs. TN	1.38 (0.37–5.01)	0.63
Number of prior lines for MBC		0.83 (0.59–1.18)	0.30
Previous CNS treatment	Yes vs. No	0.62 (0.21–1.80)	0.38
CNS therapies	WBRT vs. (SRS + neurosurgery)	1.10 (0.35–3.51)	0.87

PS: performance status; HR: hormone receptors; TN: triple-negative; MBC: metastatic breast cancer; WBRT: whole brain radiotherapy; SRS: stereotactic surgery.

**Table 3:** Overall survival (OS) cox regression univariate analysis.

OS Cox Univariate Analysis (*n* = 20)		Hazard Ratio (95CI)	*p*-Value
Age at inclusion, years		0.99 (0.96–1.03)	0.62
PS	1 vs. ≥2	0.15 (0.04–0.58)	0.006
Pathological subtype			0.27
	HR+ vs. TN	0.49 (0.16–1.53)	0.22
	HER2+ vs. TN	1.24 (0.37–4.10)	0.73
Number of prior lines for MBC		0.95 (0.69–1.31)	0.76
Previous CNS treatment	Yes vs. No	0.62 (0.21–1.80)	0.38

PS: performance status; HR: hormone receptors; TN: triple-negative; MBC: metastatic breast cancer; CNS: central nervous system.

## Data Availability

The data presented in this study are available on request from the corresponding author. The data are not publicly available due to identity reasons.

## Supplementary Materials

The following are available online at https://www.mdpi.com/2077-0383/10/6/1272/s1, Figure S1: Flowchart diagram, Table S1: STROBE Statement—checklist of items that should be included in reports of observational studies.
