# Supplementary material for: Eribulin Efficacy on Brain Metastases in Heavily Pretreated Patients with Metastatic Breast Cancer
Source: J Clin Med. 2021 Mar 18;10(6):1272. doi: 10.3390/jcm10061272 (PMC8003126; doi:10.3390/jcm10061272)
Supplement: Supplementary file 1 [file jcm-10-01272-s001.pdf]

Supplementary Materials

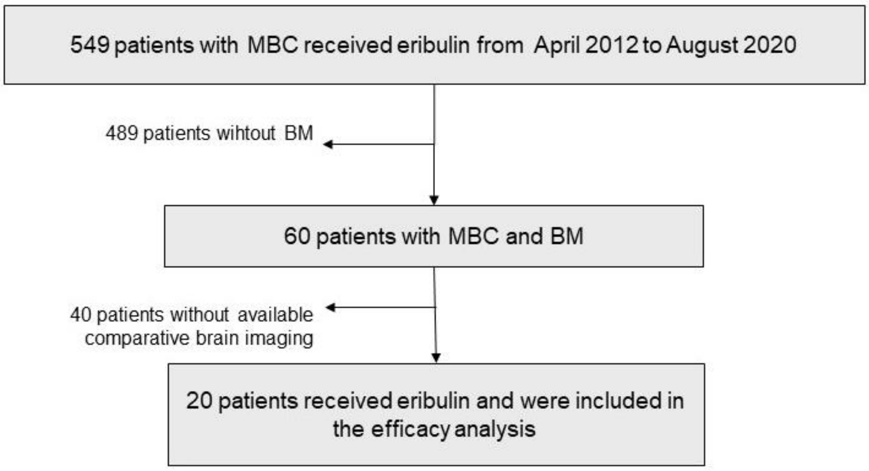

Figure S1. Flowchart diagram.

Table S1. STROBE Statement—checklist of items that should be included in reports of observational studies.

	**Item No**	**Recommendation**	**Paragraph numbers in section***
Title and abstract	1	(a) Indicate the study's design with a commonly used term in the title or the abstract	Abstract Section 2
		(b) Provide in the abstract an informative and balanced summary of what was done and what was found	Abstract Section 2–3
		**Introduction**	
Background/rationale	2	Explain the scientific background and rationale for the investigation being reported	1–2–3
Objectives	3	State specific objectives, including any prespecified hypotheses	4
		**Methods**	
Study design	4	Present key elements of study design early in the paper	Demographics and endpoints paragraphs
Setting	5	Describe the setting, locations, and relevant dates, including periods of recruitment, exposure, follow-up, and data collection	Demographics paragraph
Participants	6	Cohort study—Give the eligibility criteria, and the sources and methods of selection of participants. Describe methods of follow-up.<br>Case-control study—Give the eligibility criteria, and the sources and methods of case ascertainment and control selection. Give the rationale for the choice of cases and controls.<br>Cross-sectional study—Give the eligibility criteria, and the sources and methods of selection of participants	Demographics paragraph
		(b) Cohort study—For matched studies, give matching criteria and number of exposed and unexposed.<br>Case-control study—For matched studies, give matching criteria and the number of controls per case	

Variables	7	Clearly define all outcomes, exposures, predictors, potential confounders, and effect modifiers. Give diagnostic criteria, if applicable	Endpoint paragraph
Data sources/ measurement	8*	For each variable of interest, give sources of data and details of methods of assessment (measurement). Describe comparability of assessment methods if there is more than one group	Endpoints paragraph
Bias	9	Describe any efforts to address potential sources of bias	
Study size	10	Explain how the study size was arrived at	NA for this retrospective study
Quantitative variables	11	Explain how quantitative variables were handled in the analyses. If applicable, describe which groupings were chosen and why	Statistical analysis paragraph
Statistical methods	12	(a) Describe all statistical methods, including those used to control for confounding	Statistical analysis paragraph
		(b) Describe any methods used to examine subgroups and interactions	Statistical analysis paragraph
		(c) Explain how missing data were addressed	ND
		(d) Cohort study—If applicable, explain how loss to follow-up was addressed<br>Case-control study—If applicable, explain how matching of cases and controls was addressed<br>Cross-sectional study—If applicable, describe analytical methods taking account of sampling strategy	Statistical analysis paragraph
		(e) Describe any sensitivity analyses	
		**Results**	
Participants	13*	(a) Report numbers of individuals at each stage of study—e.g. numbers potentially eligible, examined for eligibility, confirmed eligible, included in the study, completing follow-up, and analyzed	Supplementary figure 1 (CONSORT diagram)
		(b) Give reasons for non-participation at each stage	Supplementary figure 1 (CONSORT diagram)
		(c) Consider use of a flow diagram	Supplementary figure 1 (CONSORT diagram)
Descriptive data	14*	(a) Give characteristics of study participants (e.g. demographic, clinical, social) and information on exposures and potential confounders	Demographics paragraph and Table 1
		(b) Indicate number of participants with missing data for each variable of interest	Table 1
		(c) Cohort study—Summaries follow-up time (e.g., average and total amount)	Eribulin efficacy on BCBM paragraph
Outcome data	15*	Cohort study—Report numbers of outcome events or summary measures over time	Eribulin efficacy on BCBM paragraph
		Case-control study—Report numbers in each exposure category, or summary measures of exposure	*NA*
		Cross-sectional study—Report numbers of outcome events or summary measures	NA
Main results	16	(a) Give unadjusted estimates and, if applicable, confounder-adjusted estimates and their precision (e.g., 95% confidence interval). Make clear which confounders were adjusted for and why they were included	Eribulin efficacy on BCBM paragraph, Figure 1, and table 2
		(b) Report category boundaries when continuous variables were categorized	Eribulin efficacy on BCBM paragraph and table 2
		(c) If relevant, consider translating estimates of relative risk into absolute risk for a meaningful time period	NA

Other analyses	17	Report other analyses done—e.g. analyses of subgroups and interactions, and sensitivity analyses	Other survival endpoints paragraph, Figures 2 and 3, and table 3
**Discussion**			
Key results	18	Summaries key results with reference to study objectives	1st paragraph
Limitations	19	Discuss limitations of the study, taking into account sources of potential bias or imprecision. Discuss both direction and magnitude of any potential bias	5th paragraph
Interpretation	20	Give a cautious overall interpretation of results considering objectives, limitations, multiplicity of analyses, results from similar studies, and other relevant evidence	Paragraphs 2 to 4 and conclusion
Generalizability	21	Discuss the generalizability (external validity) of the study results	Conclusion section
**Other information**			
Funding	22	Give the source of funding and the role of the funders for the present study and, if applicable, for the original study on which the present article is based	Done

*Give information separately for cases and controls in case-control studies and, if applicable, for exposed and unexposed groups in cohort and cross-sectional studies.

Note: An Explanation and Elaboration article discusses each checklist item and gives methodological background and published examples of transparent reporting. The STROBE checklist is best used in conjunction with this article (freely available on the Web sites of PLoS Medicine at http://www.plosmedicine.org/ (accessed on 12 March 2021), Annals of Internal Medicine at http://www.annals.org/ (accessed on 12 March 2021), and Epidemiology at http://www.epidem.com/ (accessed on 12 March 2021)). Information on the STROBE Initiative is available at www.strobe-statement.org (accessed on 12 March 2021).
